# Combination of Melittin and *Clinacanthus nutans* Extract Enhances Antiviral Efficacy Against Dengue Virus Serotype 2 Infection

**DOI:** 10.3390/ijms27177782

**Published:** 2026-08-31

**Authors:** Natthanich Boonsatit, Somluethai Fungfueang, Saruda Thongyim, Yingmanee Tragoolpua, Terd Disayathanoowat, George S. Baillie, Aussara Panya

**Affiliations:** 1 Master of Science Program in Applied Microbiology, Faculty of Science, Chiang Mai University, Chiang Mai 50200, Thailand; natthanich1802@gmail.com (N.B.); somluethai_fungf@cmu.ac.th (S.F.); 2 Cell Engineering for Cancer Therapy Research Group, Faculty of Science, Chiang Mai University, Chiang Mai 50200, Thailand; supawadeethongyim@gmail.com (S.T.); yingmanee.t@cmu.ac.th (Y.T.); 3 Department of Biology, Faculty of Science, Chiang Mai University, Chiang Mai 50200, Thailand; terd.dis@cmu.ac.th; 4 School of Cardiovascular and Metabolic Health, College of Veterinary Medical and Life Science, University of Glasgow, Glasgow G12 8TA, UK; george.baillie@glasgow.ac.uk

**Keywords:** dengue virus, DENV, peptide, melittin, *Clinacanthus nutans*, antiviral agent

## Abstract

Dengue virus (DENV) remains a major global health threat, particularly in tropical and subtropical regions, due to the lack of effective antiviral therapies. In this study, we evaluated a novel combination strategy using melittin, a bee venom-derived antiviral peptide, together with *Clinacanthus nutans* extract to enhance antiviral efficacy while reducing melittin-associated cytotoxicity against dengue virus serotype 2 (DENV-2). Cytotoxicity was assessed in Vero cells using a cell viability assay, whereas antiviral activity was evaluated by focus-forming unit (FFU) reduction assay, cell-based ELISA, FFU titration assay, and immunofluorescence assay (IFA). Melittin (1.25–2.5 µg/mL) and *C. nutans* extract (15.625–500 µg/mL) maintained cell viability above 80%, whereas 5 µg/mL melittin markedly reduced cell viability. Notably, co-treatment with *C. nutans* restored cell viability to above 80%, demonstrating a significant cytoprotective effect. More importantly, the combination treatment exhibited greater antiviral activity than either agent alone, resulting in complete inhibition of viral infection at lower concentrations while simultaneously suppressing intracellular viral protein expression and production of progeny viruses. These findings suggest that *C*. *nutans* may enhance the antiviral activity of melittin while reducing its cytotoxicity under the conditions tested. Overall, this study provides preliminary evidence supporting the potential of this natural compound-based combination strategy and warrants further preclinical investigation as a candidate antiviral approach against DENV infection.

## 1. Introduction

Dengue virus (DENV), a member of the *Flavivirus* genus within the *Flaviviridae* family, is the causative agent of dengue fever, a mosquito-borne disease that remains a major public health concern in tropical and subtropical regions worldwide [1,2]. DENV is estimated to cause 100–400 million infections annually, resulting in approximately 20,000–25,000 deaths each year [1,3]. The virus is primarily transmitted by infected female mosquitoes, particularly *Aedes aegypti* and *Aedes albopictus*, which are commonly found in urban and semi-urban areas [2,4]. Severe infection can lead to life-threatening complications, including Dengue Hemorrhagic Fever (DHF) and Dengue Shock Syndrome (DSS) [2,5]. Structurally, DENV is an enveloped positive-sense single-stranded RNA virus composed of three structural proteins (C, prM/M, and E) and seven non-structural proteins (NS1–NS5), which are essential for viral replication and pathogenesis [4,6,7]. Viral infection is initiated through receptor-mediated endocytosis involving host receptors such as heparan sulfate proteoglycans and C-type lectin receptors [8,9].

Current DENV prevention strategies mainly rely on vector control, including elimination of mosquito breeding sites, release of genetically modified mosquitoes, and *Wolbachia*-based approaches to reduce viral transmission [10,11]. Although vaccine development has advanced, the first licensed vaccine, CYD-TDV (Dengvaxia), showed limitations related to safety and variable efficacy among seronegative individuals [12,13]. Newer vaccine candidates, including TAK-003 and TV003/TV005, have shown improved clinical outcomes [14,15,16]. However, dengue infection remains a significant global burden, highlighting the urgent need for more effective antiviral therapies [3,16].

Bee venom (apitoxin), produced by honeybees of the *Apis* genus, contains several bioactive compounds, among which melittin is the major peptide, accounting for approximately 40–60% of the dry weight of bee venom [17]. Melittin is a cationic amphipathic peptide that exhibits broad biological activities, including antiviral effects through disruption of viral envelope structures and interference with viral replication [18,19]. Previous studies have demonstrated its antiviral activity against multiple viruses, including Influenza A virus, Human Immunodeficiency Virus (HIV), Herpes Simplex Virus (HSV), Respiratory Syncytial Virus (RSV), and Vesicular Stomatitis Virus (VSV) [19]. Despite its potent antiviral properties, melittin has limited therapeutic application due to its high cytotoxicity [18,20,21].

*Clinacanthus nutans* is a medicinal herb widely used in Southeast Asia and included in Thailand’s National List of Essential Medicines [22]. The plant possesses multiple pharmacological activities, including antiviral, anti-inflammatory, antioxidant, and immunomodulatory properties [22,23]. Traditionally, it has been used for the treatment of viral infections such as HSV and herpes zoster [23]. Previous studies have also suggested that the extract inhibits viral attachment during the early stage of infection and may suppress viral replication through interaction with the viral NS5 protein [24]. In addition, several flavonoids, including schaftoside, vitexin, isovitexin, and orientin, have been identified as major bioactive constituents and are considered to contribute to the antiviral and antioxidant activities of the plant [24]. Furthermore, *C. nutans* extract has demonstrated cytoprotective [24] and anti-inflammatory effects by suppressing pro-inflammatory genes, including *IL1β*, *IL6*, and *CXCL3*, while reducing inflammation-induced cell death [22,25]. Based on the antiviral and cytoprotective properties of *C. nutans*, this study aimed to evaluate whether combining *C. nutans* extract with melittin could enhance antiviral activity against DENV while minimizing melittin-induced cytotoxicity.

## 2. Results

### 2.1. Cytotoxicity of Melittin in Vero Cells

To evaluate cytotoxicity, Vero cells were treated with melittin at concentrations ranging from 1.25 to 20 µg/mL and incubated for 24, 48, and 72 h. Cell viability was subsequently determined by 0.1% crystal violet staining. The results demonstrated that melittin induced cell death in a dose-dependent manner, with concentrations above 5 µg/mL showing significant cytotoxic effects on Vero cells. The calculated 50% cytotoxic concentration (CC_50_) values were approximately 3.444, 4.867, and 4.303 µg/mL at 24, 48, and 72 h post-treatment, respectively (Table 1 and Figures S1 and S2).

### 2.2. Melittin Potentially Inhibits DENV-2 Infection

**Figure 1:**
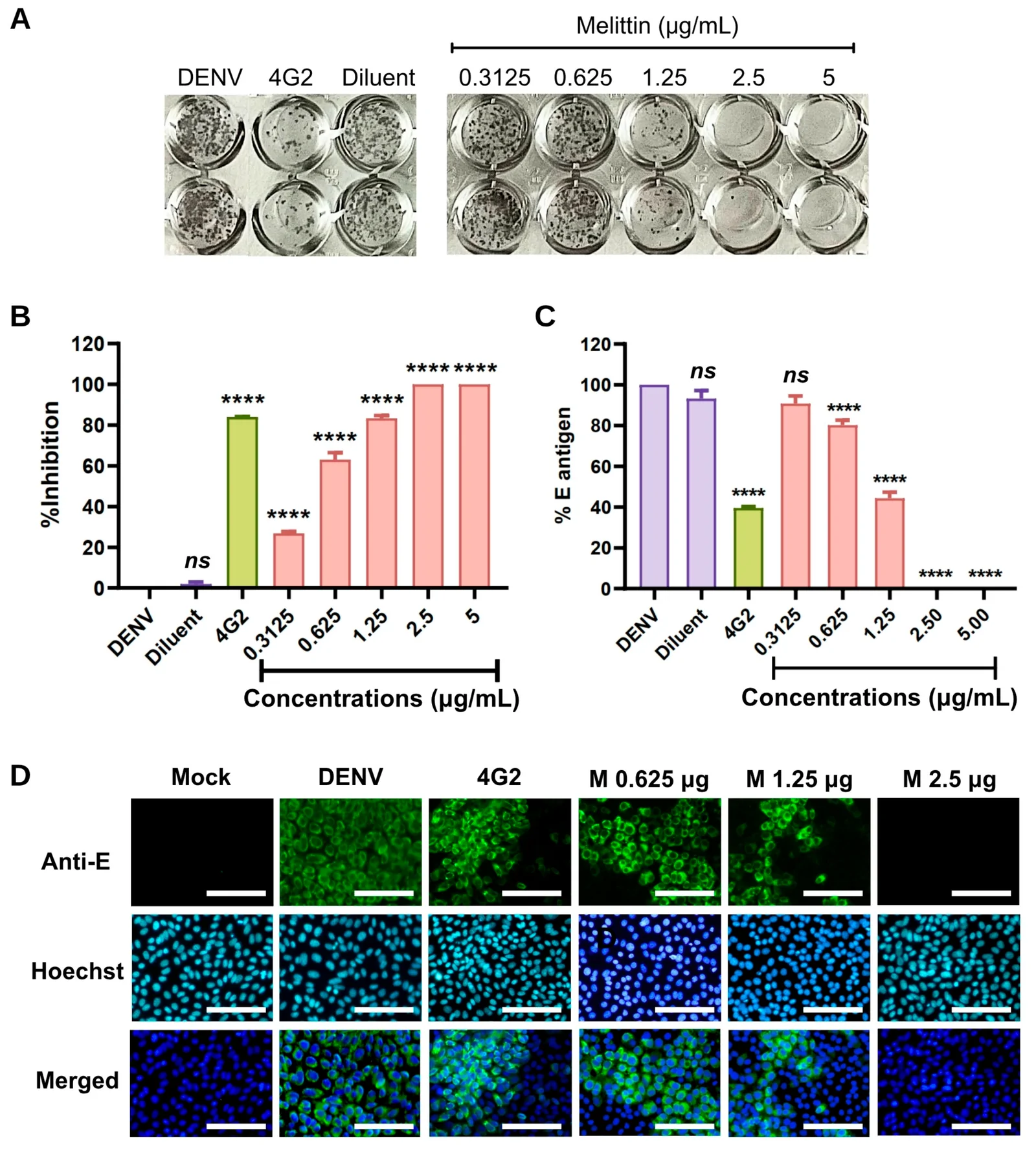
Antiviral activity of melittin against DENV-2 infection. Vero cells were infected with DENV-2 in the presence or absence of melittin (0.3125–5 µg/mL). The infected cells were harvested and stained for focus-forming units (FFU) (**A**) and used to calculate the percentage of inhibition relative to non-treated control (**B**). The percentage of intracellular E antigen (**C**), and the number of infected cells (**D**) were determined using the cell-based ELISA and immunofluorescence assay (IFA), respectively. In panel (**D**), “M” indicates melittin. The scale bar in panel (**D**) represents 50 µm. Data are presented as mean ± SEM from three independent experiments (*n* = 3). Statistical analyses for panels (**A**–**D**) were performed using one-way ANOVA followed by Tukey’s multiple comparisons test. **** *p* < 0.0001; *ns*, not significant.

The antiviral activity of melittin against DENV was evaluated using a focus-forming unit (FFU) reduction assay by quantifying viral foci relative to the untreated virus control, which was defined as 100% infection (Figure 1A,B). Melittin inhibited DENV infection in a concentration-dependent manner. Complete inhibition was observed at concentrations of 2.5–5 µg/mL, whereas treatment at 0.3125, 0.625, and 1.25 µg/mL reduced viral infection by 26.88%, 63.09%, and 83.37%, respectively (Figure 1B).

The antiviral activity of melittin was further assessed by measuring DENV protein expression using cell-based ELISA and expressed as the percentage of E antigen relative to the untreated virus control, which was defined as 100% infection. Melittin significantly reduced viral protein expression in a concentration-dependent manner. Complete inhibition was observed at concentrations of 2.5–5 µg/mL, resulting in undetectable E antigen expression (0%) (Figure 1C). At lower concentrations of 0.3125, 0.625, and 1.25 µg/mL, E antigen expression was reduced to 90.77%, 80.25%, and 44.38%, respectively (Figure 1C). Consistent with these findings, immunofluorescence assay (IFA) confirmed a marked reduction in the number of infected cells following melittin treatment (Figure 1D).

### 2.3. Docking Suggests a Possible Interaction Between Melittin and the DENV Envelope Protein

To gain insight into the potential mechanism underlying the antiviral activity of melittin, molecular docking analysis was performed to evaluate its predicted interaction with the DENV envelope protein. Given the reported membrane-active properties of melittin, we hypothesized that direct interaction with viral surface proteins may contribute to its antiviral activity. Therefore, molecular docking was used to assess the potential binding of melittin to the DENV envelope protein.

**Figure 2:**
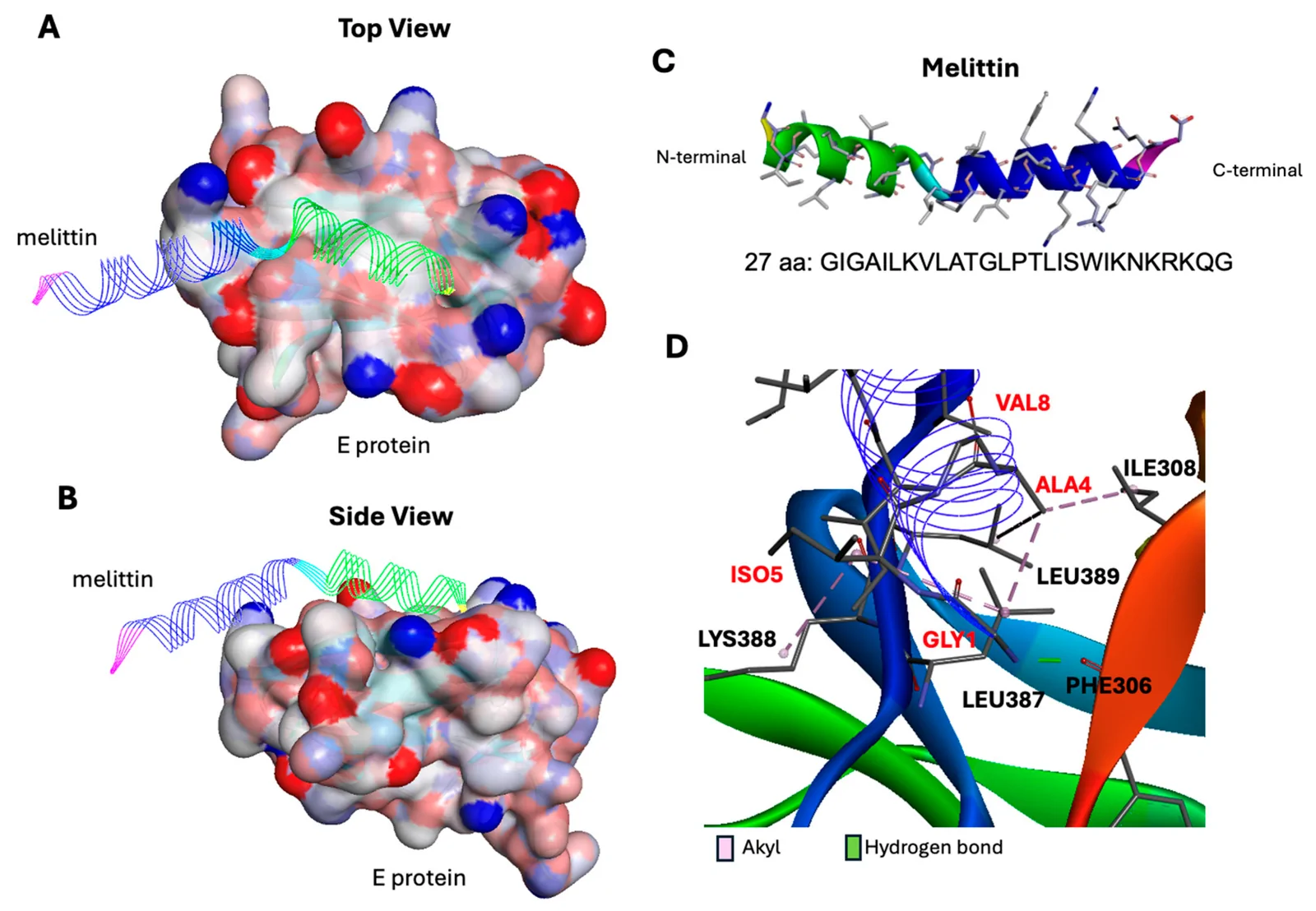
Predicted interaction between melittin and the DENV-2 envelope protein. Molecular docking analysis was performed using the ZDOCK program to investigate the interaction between melittin (rainbow ribbon) and domain III of the DENV envelope protein (surface shape) (**A**,**B**). The docking results revealed that melittin (**C**) predominantly interacted with the domain III residues (black-colored residues) through its N-terminal region (red-colored residues) (**D**).

The docking analysis predicted that melittin predominantly interacted through its N-terminal hydrophilic region with domain III of the DENV envelope protein with ZDOCK score of −21.288 (Figure 2A,B). Specifically, melittin formed alkyl interactions with ILE308, LEU387, and LEU389, hydrogen bonding with PHE307, and additional van der Waals interactions with LYS305, LYS307, ILE312, TYR377, GLY385, GLN386, ASN390, and LYS394 (Figure 2C,D). These findings suggest a potential interaction between melittin and domain III of the DENV envelope protein. However, this predicted interaction remains to be experimentally validated and warrants further investigation.

### 2.4. Protective Effect of Clinacanthus nutans Against Melittin-Induced Cytotoxicity

**Figure 3:**
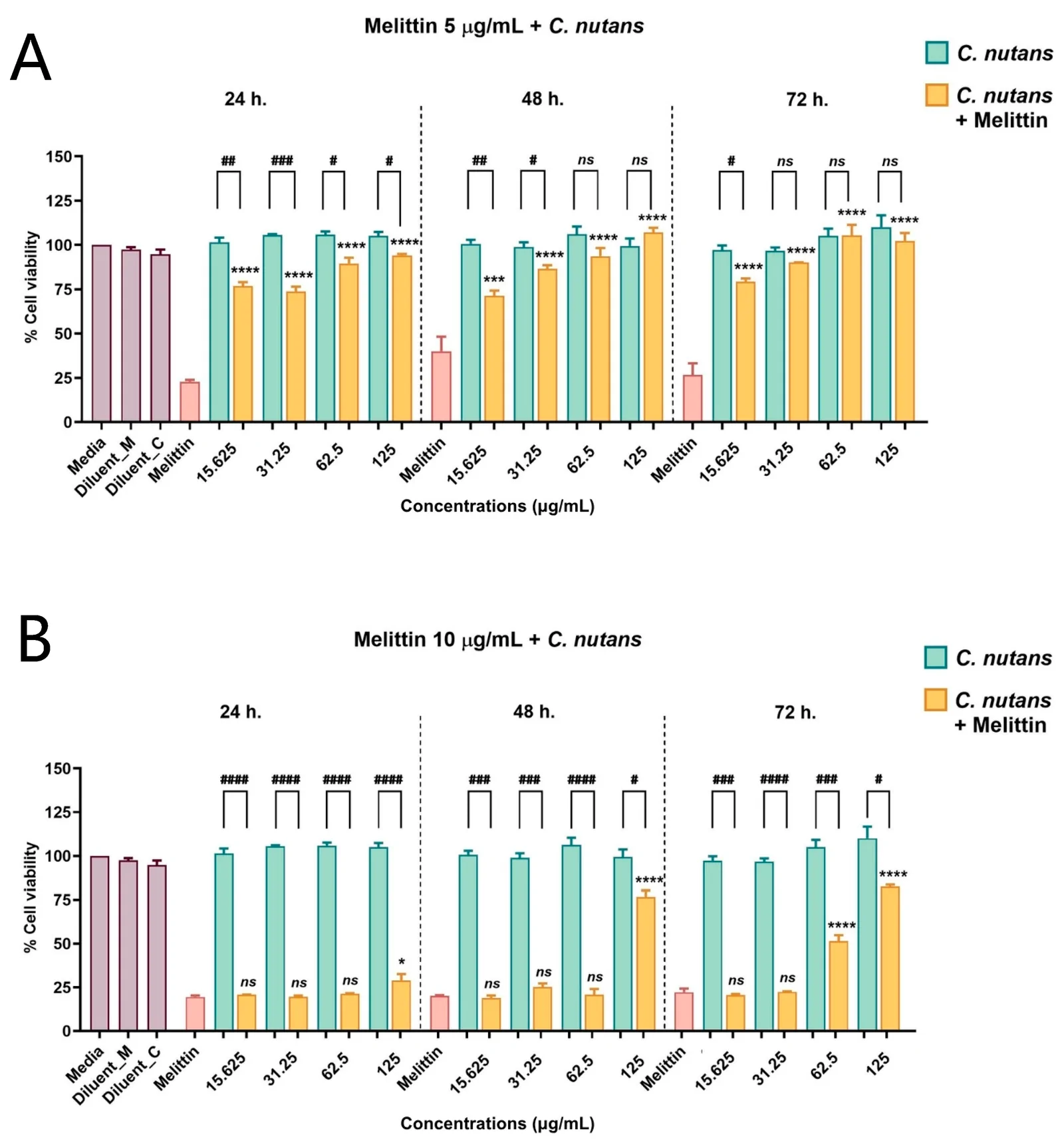
*C. nutans* extract attenuated melittin-induced cytotoxicity. Combination treatment was evaluated using *C. nutans* extract at concentrations ranging from 7.8125 to 125 µg/mL in combination with melittin at 5 µg/mL (**A**) or 10 µg/mL (**B**). Diluent_M refers to the diluent used for melittin, whereas Diluent_C refers to the diluent used for *C. nutans* extract. Cell viability was determined at 24, 48, and 72 h after treatment. Data are presented as mean ± SEM from three independent experiments (*n* = 3). Statistical analysis was performed using one-way ANOVA followed by Tukey’s multiple comparisons test. *ns*, not significant; * *p* < 0.05, *** *p* < 0.001, **** *p* < 0.0001 versus melittin alone; # *p* < 0.05, ## *p* < 0.01, ### *p* < 0.001, and #### *p* < 0.0001 versus *C. nutans* extract alone.

Although melittin exhibited potent antiviral activity against DENV, its therapeutic application was limited by significant cytotoxicity observed at 5 µg/mL. Therefore, the protective effect of *C. nutans* against melittin-induced cytotoxicity was further investigated. *C. nutans* extract alone showed low cytotoxicity toward Vero cells, with no significant reduction in cell viability at concentrations up to 250 µg/mL (Figure S2). The combination treatment was evaluated using melittin at concentrations ranging from 1.25 to 20 µg/mL together with different concentrations of *C. nutans* extract, and the complete results are shown in Figure S3. Based on these data, the effects of the combination using melittin at 5 and 10 µg/mL together with *C. nutans* extract at concentrations ranging from 7.8125 to 125 µg/mL are presented in Figure 3.

At 5 µg/mL melittin, co-treatment with *C. nutans* significantly improved cell viability in a concentration-dependent manner. Cell viability increased from 71.3441% at 15.625 µg/mL to 86.6221% at 31.25 µg/mL after 48 h, while higher concentrations (62.5–125 µg/mL) almost completely restored cell viability across all time points (Figure 3A). At the higher melittin concentration (10 µg/mL), protective effects were observed only at elevated *C. nutans* concentrations, where 62.5 µg/mL increased cell viability to 51.3% at 72 h and 125 µg/mL restored viability above 80% after 48–72 h (Figure 3B). Collectively, these findings demonstrate that *C. nutans* effectively attenuated melittin-induced cytotoxicity and significantly improved cell survival in a concentration-dependent manner.

### 2.5. Antiviral Activity of C. nutans and Combination Treatment with Melittin Against DENV

**Figure 4:**
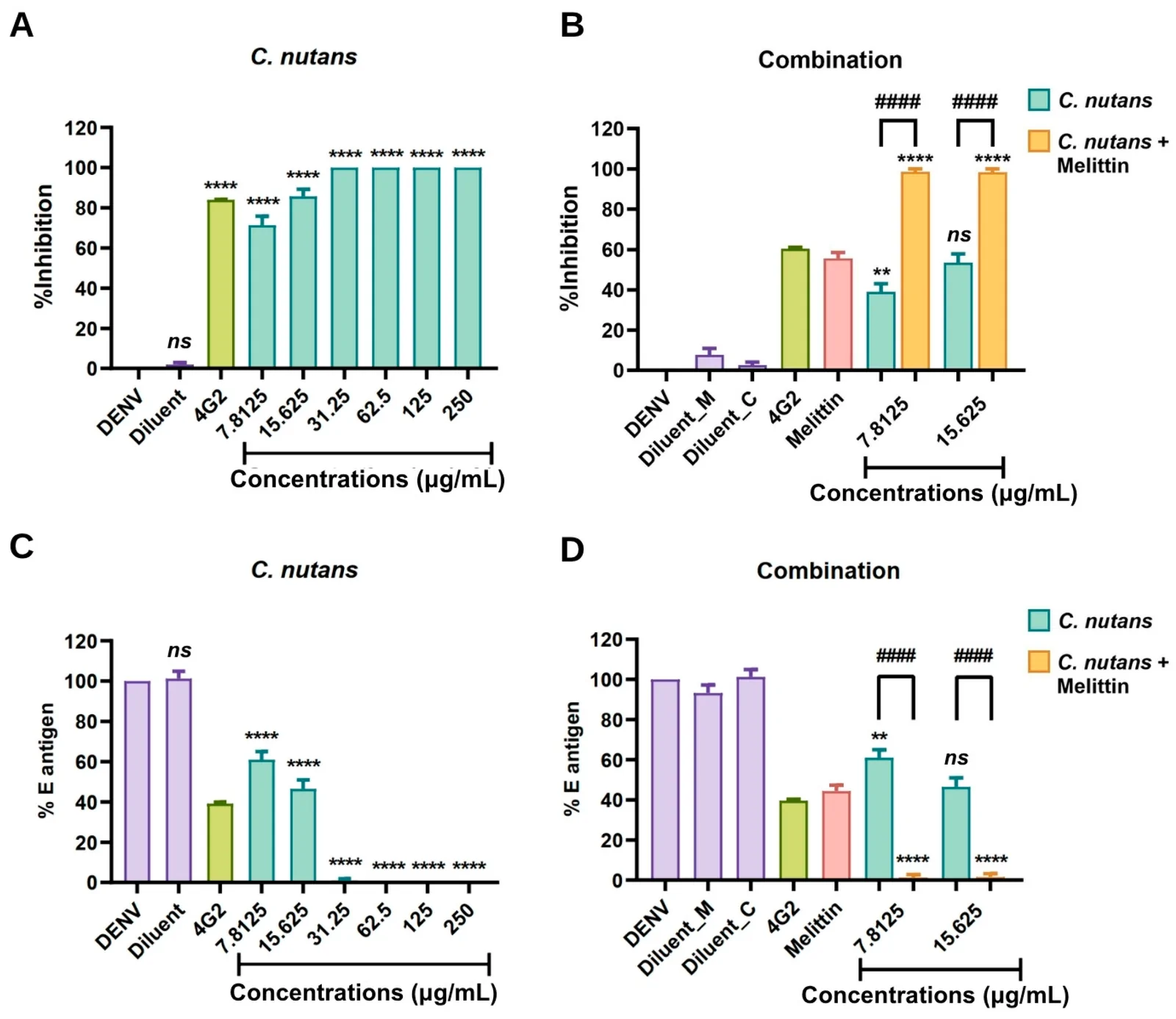
Combined *C. nutans* extract and melittin improved the antiviral activity against DENV-2 infection. The antiviral activity of *C. nutans* extract alone or in combination with melittin was evaluated using the FFU reduction assay (**A**,**B**) and cell-based ELISA (**C**,**D**). Diluent_M refers to the diluent used for melittin, whereas Diluent_C refers to the diluent used for *C. nutans* extract. Data are presented as mean ± SEM from three independent biological replicates (FFU reduction assay) or four independent biological replicates (cell-based ELISA). Statistical analysis for panels (**A**–**D**) was performed using one-way ANOVA followed by Tukey’s multiple comparisons test. *ns*, not significant; ** *p* < 0.01 and **** *p* < 0.0001 versus the DENV-2-infected control; #### *p* < 0.0001 versus the corresponding *C. nutans* extract alone.

The antiviral activity of *C. nutans* extract against DENV was further evaluated using the FFU reduction assay. *C. nutans* extract exhibited potent antiviral activity in a concentration-dependent manner, achieving complete inhibition (100%) at concentrations ranging from 31.25 to 250 µg/mL (Figure 4A). At lower concentrations of 7.8125, and 15.625 µg/mL, viral inhibition was observed at 71.38%, and 85.72%, respectively (Figure 4A). Notably, combination treatment consisting of melittin (1.25 µg/mL) together with *C. nutans* extract at concentrations of 7.8125 to 15.625 µg/mL resulted in complete viral inhibition (100%), showing significantly enhanced antiviral activity compared with individual treatments alone (Figure 4B).

Furthermore, cell-based ELISA further confirmed the antiviral activity of *C. nutans* extract and combination treatment by measuring viral E protein expression. *C. nutans* extract at concentrations of 31.25–125 µg/mL completely inhibited viral protein production, resulting in undetectable E antigen expression (0%) (Figure 4C). Notably, combination treatment with melittin (1.25 µg/mL) and *C. nutans* extract at concentrations of 7.8125 and, 15.625 µg/mL reduced E antigen expression to 1.38% and, 1.60%, respectively (Figure 4D). Statistical analysis demonstrated that combination treatment significantly improved the antiviral activity against DENV-2 infection.

### 2.6. Synergistic Antiviral Activity of C. nutans and Combination Treatment with Melittin Against DENV

The interaction between melittin and *C. nutans* extract was evaluated using the Bliss independence model. Bliss analysis was performed separately for viral inhibition measured by the focus-forming unit (FFU) reduction assay and residual viral E antigen measured by ELISA. Because higher concentrations of the individual treatments and their combinations produced nearly complete antiviral inhibition, only two low-dose concentration pairs were suitable for interaction analysis. Bliss independence analysis of the FFU reduction assay showed positive Bliss synergy scores (ΔBliss) of 0.257 and 0.190 for the combinations of melittin (1.25 µg/mL) with *C. nutans* extract at 7.8125 and 15.625 µg/mL, respectively. Consistent with these findings, synergy factor (SF) analysis based on residual viral E antigen levels measured by ELISA yielded SF values of 19.615 and 12.588 for the corresponding combinations. The positive ΔBliss values together with SF values greater than 1 suggest that these two tested low-dose combinations exhibited synergistic interactions under the experimental conditions (Table 2).

### 2.7. Selectivity Index and Apparent Therapeutic Window

To further assess the therapeutic window of each treatment, the 50% effective concentration (EC_50_), CC_50_, and selectivity index (SI) were calculated. The reported CC_50_ values were obtained from the 72-h cell viability assay. Melittin exhibited an EC_50_ of 1.077 µg/mL (95% confidence interval [CI]: 0.9795 to 1.180) and a CC_50_ of 4.303 µg/mL (95% CI: 3.411 to 5.867), resulting in an SI of 3.99. In comparison, *C. nutans* extract showed an EC_50_ of 11.31 µg/mL (95% CI: 9.628 to 13.06) and a CC_50_ of 479.7 µg/mL, resulting in an SI of 42.4.

The SI of the combination treatment could not be calculated because all tested concentration pairs produced complete inhibition of DENV infection, precluding reliable estimation of the EC_50_ from a conventional dose–response curve. In addition, a reliable CC_50_ for the combination treatment could not be determined from the tested concentration pairs. Therefore, the SI and therapeutic window of the combination treatment could not be quantitatively determined.

### 2.8. The Bioactive Compounds of C. nutans

Liquid chromatography–tandem mass spectrometry (LC-MS/MS) analysis was performed to characterize the phytochemical composition of *C. nutans* extract and identify bioactive compounds potentially associated with its antiviral activity. Compounds with a library match score of ≥95% were considered putatively identified and are summarized in Table 3 and Table S1. In total, 29 putatively identified compounds were detected (Table S1), of which 7 have previously been reported to possess antimicrobial activity including acteoside (C_29_H_36_O_15_), Isovitexin (C_21_H_20_O_10_), Schaftside (C_26_H_28_O_14_), *p*-Coumaric acid (C_9_H_8_O_3_), Linoleic acid (C_18_H_32_O_2_), Adenosine (C_10_H_13_N_5_O_4_) (Table 3). In addition, 12 putatively identified compounds have been reported to be involved with cell proliferative. These compounds may contribute to the biological activities of the extract; however, their individual roles in the antiviral and cytoprotective effects observed in this study remain to be experimentally validated.

## 3. Discussion

The development of effective anti-DENV therapeutics remains urgently needed to reduce mortality associated with severe DENV infection and to control viral transmission during epidemic outbreaks. In the present study, melittin exhibited potent antiviral activity against DENV, while combination treatment with *C. nutans* further enhanced its inhibitory effect against DENV infection in Vero cells.

Melittin, the major peptide constituent of *Apis mellifera* venom, accounts for approximately 50% of the total dry weight of bee venom and is largely responsible for its biological activity. Due to its amphipathic structure, melittin possesses broad biological activities and has been extensively reported to exhibit antimicrobial, antiviral, anti-inflammatory, immunomodulatory, and anticancer properties [18,19]. Recent studies have further demonstrated that melittin can induce apoptosis and suppress tumor progression through modulation of multiple cellular signaling pathways [21]. In addition, its membrane-disruptive activity allows direct interaction with lipid bilayers and pore formation in both cellular and viral membranes, which largely contributes to its broad antimicrobial and antiviral efficacy [21]. Despite these promising pharmacological properties, the therapeutic application of melittin remains limited by dose-dependent cytotoxicity at high concentrations [52,53].

Consistent with previous reports, melittin reduced Vero cell viability in a concentration-dependent manner, with concentrations of 5–20 µg/mL markedly decreasing cell survival. This cytotoxicity has been widely attributed to its strong membrane-disruptive properties [21,53]. Previous studies have shown that excessive melittin exposure can disrupt cellular membranes and activate phospholipase A2, resulting in calcium influx and subsequent activation of inflammatory signaling pathways, including NF-κB-mediated cytokine production [25,52]. Under these conditions, melittin has been reported to stimulate the release of pro-inflammatory mediators, including IL-1β and IL-6, which may further exacerbate cellular damage and tissue inflammation [52,53]. Interestingly, combination treatment significantly improved cell viability compared with melittin alone, suggesting that *C. nutans* may protect against melittin-associated cytotoxicity. This cytoprotective effect may be associated with the previously reported anti-inflammatory properties of *C. nutans*, which have been shown to suppress pro-inflammatory mediators, including *IL-1β*, *IL-6*, and *CXCL3* [21,39]. Collectively, these findings suggest that *C. nutans* may mitigate melittin-induced toxicity and improve the therapeutic potential of melittin as a candidate antiviral agent.

The observed antiviral activity may be associated with the membrane-active amphipathic structure of melittin, which has previously been reported to disrupt viral membranes through pore formation and contribute to viral inactivation [18,53]. The antiviral assay demonstrated that melittin effectively inhibited DENV infection following pre-incubation of the virus with the test sample. In addition, molecular docking analysis predicted a potential interaction between melittin and domain III of the DENV envelope protein. Taken together, these findings suggest that melittin may interfere with an early stage of DENV infection. However, neither the antiviral assay nor the docking analysis can determine the precise mechanism of viral inhibition. The docking analysis is based on a rigid-body computational model and should be considered a computational prediction rather than experimental evidence of direct binding. Therefore, other mechanisms may also contribute to the observed antiviral activity, including disruption of the viral envelope through melittin’s membrane-lytic activity, viral particle aggregation, nonspecific membrane perturbation, or interference with viral adsorption. Further mechanistic studies, such as time-of-addition, virucidal, viral binding, viral entry, and viral particle integrity assays, will be necessary to determine the precise mechanism by which melittin inhibits DENV infection.

Importantly, the combination treatment showed greater antiviral activity than either melittin or *C. nutans* extract alone. To further evaluate the interaction between the two agents, Bliss independence analysis and SF analysis were performed. The observed antiviral inhibition exceeded the inhibition predicted by the Bliss independence model for both combination pairs, resulting in positive ΔBliss. In addition, SF analysis based on residual viral E antigen levels yielded values greater than 1 for both combinations. The enhanced antiviral activity may be explained by the complementary antiviral properties of the two agents; however, this interpretation remains hypothetical. Melittin has been reported to directly disrupt viral particles and inhibit viral attachment [18,53], whereas *C. nutans* extract interferes with viral infection during the early stage of infection and may inhibit viral replication through its interaction with the viral NS5 protein [24]. Based on these previous findings, it is possible that the combination affects DENV infection through multiple mechanisms, which may contribute to the enhanced antiviral activity observed in this study. However, the present study did not determine the specific stage of viral inhibition or the precise mechanism responsible for the observed antiviral effect. Notably, changing the concentration of either agent may alter the overall antiviral response. At certain concentration combinations, the two agents may work more effectively together, resulting in greater viral inhibition, whereas other combinations may produce a weaker effect. In addition, *C. nutans* extract contains multiple bioactive compounds, and the relative contribution of these constituents may vary with concentration. Therefore, the observed synergistic interaction should be interpreted only within the tested concentration range. Future studies using lower concentration ranges and a more comprehensive dose–response matrix are needed to determine whether this interaction is maintained across a broader range of concentration combinations.

The findings of the present study suggest that the combination of melittin and *C. nutans* extract has potential as an anti-DENV strategy. Combination-based antiviral therapy has been increasingly recognized as a potential strategy to enhance therapeutic efficacy while reducing the adverse effects associated with high-dose single-agent treatment [54]. Although the combination showed potent antiviral activity at the tested concentration pairs, the therapeutic window of the combination could not be quantitatively determined. The EC_50_ could not be reliably estimated because all tested concentration pairs produced complete inhibition of DENV infection. In addition, a reliable CC_50_ for the combination could not be determined from the tested concentration pairs, and therefore the SI could not be calculated. Thus, the observed reduction in cytotoxicity at selected combination concentrations should be interpreted as a finding under the tested conditions rather than as definitive evidence of an expanded therapeutic window. In comparison, the individual-compound analyses showed that melittin exhibited potent antiviral activity but a relatively low SI (3.99), indicating a narrow margin between antiviral efficacy and cytotoxicity. In contrast, *C. nutans* extract showed a substantially higher SI (42.4), consistent with its lower cytotoxicity and wider safety margin. Taken together, the combination showed promising antiviral activity and reduced cytotoxicity at selected concentrations compared with melittin alone. However, further studies using a broader and systematic concentration matrix are required to establish reliable dose–response relationships, determine the EC_50_ and CC_50_ of the combination, and more accurately evaluate its therapeutic window.

LC–MS/MS analysis identified multiple bioactive constituents in *C. nutans* extract, several of which have previously been reported to possess antiviral, antioxidant, and anti-inflammatory properties. Although the present study was not designed to determine which individual compounds were primarily responsible for the observed antiviral and cytoprotective activities, these phytochemicals may act cooperatively to reduce cellular damage while preserving the antiviral activity of melittin. The multitarget nature of herbal extracts may therefore contribute to both the enhanced antiviral efficacy and the improved therapeutic window observed in the combination treatment. Nevertheless, further studies using bioactivity-guided fractionation and purified compounds will be required to identify the principal active constituents and their molecular targets. In addition, the LC–MS/MS analysis in the present study was used for qualitative characterization of the extract, and quantitative standardization of major marker compounds was not performed. Future studies should include extract standardization, such as high-performance liquid chromatography (HPLC) fingerprint analysis or quantitative determination of major marker compounds, to improve the reproducibility and quality control of the extract.

Peptide-based therapeutics have emerged as promising antiviral agents owing to their high target specificity, favorable safety profile, and relatively cost-effective production. In our previous studies, we successfully identified several peptide inhibitors targeting DENV, demonstrating that rationally designed peptides can effectively inhibit viral attachment and suppress DENV infection, further supporting the feasibility of peptide-based therapeutics for DENV treatment [54,55,56]. These findings collectively provide proof of concept that peptide-derived antiviral agents represent a promising strategy for anti-DENV drug development. Nevertheless, the clinical translation of melittin remains challenging due to the inherent instability of peptide-based molecules under physiological conditions. In particular, peptide degradation within the gastrointestinal tract may compromise structural integrity and reduce therapeutic efficacy [57]. Therefore, alternative delivery strategies, including injectable formulations, liposomal encapsulation, or nanoparticle-based delivery systems, may be required to maximize the therapeutic potential of melittin for future antiviral applications [58].

Given that both compounds primarily act during the early stage of viral infection, this therapeutic strategy is likely to be most effective during the initial phase of DENV infection before extensive viral replication occurs. In clinical settings, viremia is often reduced by the time patients present at the hospital, as peak viremia generally occurs during the early febrile phase of infection [4,59]. However, the possibility of persistent viral reservoirs within infected tissues cannot be excluded due to the broad tissue tropism of DENV, which has been reported to infect immune cells, endothelial cells, and other peripheral tissues [60]. Considering this dynamic infection scenario, although circulating viral load may decline, the combination of melittin and *C. nutans* may still be beneficial by suppressing newly synthesized viral particles, thereby potentially reducing the risk of disease progression. Nevertheless, the therapeutic window remains critical, as earlier administration would likely provide greater protection against severe disease development [4,61].

In addition to therapeutic application, this combination may also have potential as a preventive antiviral strategy, particularly during seasonal outbreaks in endemic regions. The relatively low production cost of peptide-based therapeutics and herbal extracts represents an important advantage, especially for DENV-endemic areas that are predominantly located in low- and middle-income countries, where access to advanced antiviral therapies remains limited [57,62]. Overall, these findings provide a strong foundation for the future development of combination-based antiviral therapy against DENV and support further preclinical investigation of natural compound-derived antiviral agents. Notably, the antiviral activity of the combined treatment in this study was evaluated only in vitro, and further validation in animal models and clinical studies is required to confirm its efficacy and safety. In addition, further mechanistic studies, including time-of-addition, viral binding, viral entry, virucidal activity, and viral particle integrity assays, are needed to clarify the precise mechanism of antiviral action. The optimal dosing regimen and long-term safety profile of the combined treatment require further investigation. In addition, because melittin is a bee venom-derived peptide with known allergenic and cytotoxic potential, comprehensive safety evaluations are necessary before considering clinical application.

## 4. Materials and Methods

### 4.1. Cell Culture

African green monkey kidney cells (Vero cells) were cultured in Minimum Essential Medium (MEM) supplemented with 10% fetal bovine serum (FBS) and 2 mM L-glutamine. Cells were maintained at 37 °C in a humidified incubator containing 5% CO_2_ and routinely subcultured every 2 days or when reaching 80–90% confluency. For passaging, cells were washed with phosphate-buffered saline (PBS), detached using 0.1% trypsin-EDTA, and resuspended in fresh complete medium for subsequent experiments.

### 4.2. Melittin

Melittin peptide, composed of 27 amino acids (GIGAILKVLATGLPTLISWIKNKRKQG), was commercially synthesized by GenScript (Nanjing, China) with a purity of ≥98%, as qualified via both mass spectrometry (MS) and HPLC analyses. The peptide was reconstituted in sterile water and stored at −70 °C until further use.

### 4.3. Virus Propagation

Dengue virus serotype 2 (DENV-2 strain Thailand/16681/84 [NCBI: txid31634]) was kindly provided by Prof. Pa-thai Yenchitsomanus, Faculty of Medicine Siriraj Hospital, Mahidol University, Thailand. The virus was propagated in *Aedes albopictus* C6/36 cells (Japan Cell Bank, Tsukuba, Ibaraki, Japan), and culture supernatants containing viral particles were collected at 5 days post-infection. Viral stocks were aliquoted and stored at −70 °C until use. A single batch was prepared and used throughout all experiments to minimize batch-to-batch variation.

### 4.4. Preparation of Clinacanthus nutans Extract

The preparation of *C. nutans* extract was performed according to the method previously described by Thongyim et al. [63]. Fresh leaves were washed, dried in a hot air oven at 60 °C for 48–72 h, and ground into powder. The dried powder was extracted with 95% ethanol at a ratio of 1:20 (*w*/*v*) under continuous shaking at room temperature for 72 h, with solvent replacement every 24 h. The extract was then filtered through Whatman No. 1 filter paper, concentrated using a rotary evaporator at 45 °C, and further dried completely before storage at −20 °C until use. A single batch of the ethanolic extract was used throughout all experiments to minimize batch-to-batch variation. The quality control data for the *C. nutans* extract used in this study, including extract standardization and phytochemical characterization, are summarized in Table S2, based on our previous publication [63].

### 4.5. Cell Viability Assay

Vero cells were seeded into 96-well plates at a density of 2 × 10^4^ cells/well and incubated overnight at 37 °C in a humidified incubator containing 5% CO_2_. Cells were then treated with melittin (0.3125–20 µg/mL), *C. nutans* extract (15.625–500 µg/mL), or combination treatment consisting of melittin (1.25–20 µg/mL) together with *C. nutans* extract (7.8125–125 µg/mL). Untreated cells cultured in complete medium were used as the negative control, while medium containing extraction solvent was included as the diluent control.

Following incubation for 24, 48 and 72 h, cell viability was determined using crystal violet staining. Briefly, culture medium was removed, and cells were stained with 0.1% crystal violet solution for 15–30 min at room temperature. Excess stain was removed by washing with distilled water, and the bound dye was dissolved in 70% ethanol. Absorbance was measured at 592–595 nm using a microplate spectrophotometer (Hangzhou Allsheng, Hangzhou, China). Cell viability was calculated using the following equation:

Cell viability (%) = (OD_570_ of treated cells/OD_570_ of untreated control cells) × 100

### 4.6. Focus-Forming Unit (FFU) Titration Assay

Vero cells were seeded in 96-well plates at 2 × 10^4^ cells/well and incubated overnight at 37 °C with 5% CO_2_. DENV was serially diluted 10-fold (10^−1^–10^−8^) in MEM supplemented with 2% FBS, added to the cells, and incubated for 2 h to allow viral adsorption. After removal of unbound virus, cells were overlaid with 1.5% carboxymethylcellulose in MEM containing 2% FBS and further incubated for 72 h.

Cells were then fixed with 3.6% formaldehyde, permeabilized with 0.2% Triton X-100, and incubated with 4G2 monoclonal antibody, followed by horseradish peroxidase (HRP)-conjugated rabbit anti-mouse IgG secondary antibody (Dako, Santa Clara, CA, USA). Foci were visualized using 3,3′-diaminobenzidine (DAB) substrate solution containing hydrogen peroxide and nickel chloride. Viral foci were counted under an inverted microscope (Drawell BDS400; Shanghai Drawell Scientific Instrument Co., LTd., Shanghai, China), and viral titer was calculated as focus-forming units per milliliter (FFU/mL).

### 4.7. Focus-Forming Unit (FFU) Reduction Assay

Vero cells were seeded in 96-well plates at a density of 2 × 10^4^ cells/well and incubated overnight at 37 °C in a humidified incubator containing 5% CO_2_. Test samples included melittin (0.3125–20 µg/mL), *C. nutans* extract (7.8125–250 µg/mL), and combination treatment consisting of melittin (1.25 µg/mL) together with *C. nutans* extract (7.8125–125 µg/mL). DENV was prepared at approximately 4000 FFU/mL, and 100 µL viral suspension (200 FFU/well) was mixed with each test sample and incubated at room temperature for 30 min prior to infection. The 4G2 monoclonal antibody was used as the positive control, the extraction solvent served as the diluent control. The virus-sample mixture was then added to cells and incubated for 2 h to allow viral adsorption. After removal of unbound virus, cells were overlaid with 1.5% carboxymethylcellulose in MEM supplemented with 2% FBS and incubated for 72 h. Viral foci were subsequently stained according to the protocol described previously.

### 4.8. Cell-Based Enzyme-Linked Immunosorbent Assay (Cell-Based ELISA)

Vero cells were seeded in 96-well plates at a density of 2 × 10^4^ cells/well and incubated overnight at 37 °C in a humidified incubator containing 5% CO_2_. Test samples were mixed with DENV at the indicated concentrations and incubated at room temperature for 30 min prior to infection. The virus-sample mixture was then added to the cells and incubated for 2 h to allow viral adsorption. After removal of unbound virus, cells were washed once and further incubated in MEM supplemented with 2% FBS for 72 h.

Cells were subsequently fixed with 3.6% formaldehyde, permeabilized with 0.2% Triton X-100, and blocked with 1% bovine serum albumin (BSA). Viral envelope (E) protein expression was detected using 4G2 monoclonal antibody followed by horseradish peroxidase (HRP)-conjugated rabbit anti-mouse IgG secondary antibody (Dako, Santa Clara, CA, USA). Tetramethylbenzidine (TMB; Invitrogen, Carlsbad, CA, USA) was used as the substrate, and the reaction was stopped using 2 N sulfuric acid. Absorbance was measured at 450 nm using a microplate spectrophotometer. The percentage of viral E antigen expression was calculated relative to that of non-treatrd control (set as 100%).

### 4.9. Immunofluorescence Assay (IFA)

Vero cells were seeded in 96-well plates at a density of 2 × 10^4^ cells/well and incubated overnight at 37 °C in a humidified incubator containing 5% CO_2_. Test samples were mixed with DENV at the indicated concentrations and incubated at room temperature for 30 min prior to infection. The virus-sample mixture was then added to the cells and incubated for 2 h to allow viral adsorption. After removal of unbound virus, cells were washed once and further incubated in MEM supplemented with 2% FBS for 72 h.

Cells were subsequently fixed with 3.6% formaldehyde, permeabilized with 0.2% Triton X-100, and blocked with 1% bovine serum albumin (BSA). Viral antigen expression was detected using 4G2 monoclonal antibody followed by Alexa Fluor 488-conjugated goat anti-mouse IgG secondary antibody (Invitrogen, Carlsbad, CA, USA; 1:1000 dilution). Cell nuclei were counterstained with Hoechst 33342 (Invitrogen, Carlsbad, CA, USA; 1:3000 dilution). Fluorescent signals were visualized and captured using an inverted fluorescence microscope (Nikon Corporation, Tokyo, Japan).

### 4.10. LC-MS/MS

The phytochemical composition of *C. nutans* crude extract was analyzed using liquid chromatography coupled with quadrupole time-of-flight mass spectrometry (LC-MS/QTOF; TripleTOF^®^ 6600+ System, SCIEX, Marlborough, MA, USA) through the analytical service of Mahidol University Frontier Research Facility: MU-FRF, Mahidol University, Thailand. Phytochemical profiling of *C. nutans* extract was performed using an Ultimate 3000 UHPLC system (Thermo Dionex, Sunnyvale, CA, USA) coupled to a TripleTOF^®^ 6600 hybrid quadrupole time-of-flight mass spectrometer (SCIEX, Framingham, MA, USA) equipped with a DuoSpray™ ion source. Briefly, 2.0 μL of sample was injected in partial-loop mode. Chromatographic separation was carried out at 35 °C with the autosampler maintained at 10 °C. Mobile phase A consisted of 0.1% formic acid in water, whereas mobile phase B consisted of 0.1% formic acid in acetonitrile. The mobile phase was delivered at a flow rate of 300 μL/min using the following gradient: 1% B (0–1.0 min), linearly increased to 95% B (1.0–11.0 min), maintained at 95% B until 15.0 min, and returned to 1% B (15.0–15.5 min), followed by column re-equilibration.

Mass spectrometric analysis was performed in both positive and negative electrospray ionization (ESI) modes. Data-dependent acquisition (DDA) was employed to acquire MS/MS spectra for precursor ions with *m*/*z* >100 and signal intensities exceeding 100 counts per second (cps). Compound annotation was performed by library matching, and only compounds with MS/MS spectral library score ≥95% were considered putatively identified and included in subsequent analyses.

### 4.11. Bliss Independence Analysis

The interaction between melittin and *C. nutans* extract was evaluated using the Bliss independence model [64]. Bliss analysis was performed separately for two experimental endpoints: viral inhibition measured by the FFU reduction assay and residual viral E antigen measured by ELISA.

For the FFU reduction assay, viral inhibition values (%) were converted to fractional values ranging from 0 to 1. The expected inhibitory effect of the combination was calculated using the following equation:

*Y*_ab,predicted_ = *Y*_a_ + *Y*_b_ − (*Y*_a_ ⋅ *Y*_b_)

where *Y*_a_ and *Y*_b_ represent the fractional inhibition produced by melittin and *C. nutans* extract alone, respectively, at the corresponding concentration. The observed fractional inhibition of the combination (*Y*_observed_) was then compared with the Bliss-predicted value. The bliss synergy score (ΔBliss) was calculated as

ΔBliss = *Y*_observed_ − *Y*_ab,predicted_

where *Y*_observed_ is the observed inhibition of the combination treatment. A positive ΔBliss value (>0) indicates a synergistic interaction, a value equal to zero (=0) indicates an additive interaction, and a negative value (<0) indicates an antagonistic interaction [64].

For the ELISA assay, residual viral E antigen levels (%) were normalized to the untreated virus control, which was defined as 100%. Synergy was evaluated by calculating the synergy factor (SF) as the ratio of the expected residual E antigen level to the observed residual E antigen level:

*S*_ab,expected_ = *S*_a_ ⋅ *S*_b_

where *S*_a_ and *S*_b_ represent the fractional residual viral E antigen levels after treatment with melittin and *C. nutans* extract alone, respectively.

SF = *S*_ab,expected_/*S*_observed_

Here, *S*_observed_ is the observed residual E antigen level after combination treatment. An SF value greater than 1 indicates synergy; an SF value of 1 indicates an additive effect; and an SF value less than 1 indicates antagonism [65].

The two analyses were performed independently because viral inhibition and residual viral E antigen were measured using different assays. All data were converted to fractional values before analysis.

### 4.12. Molecular Docking

Molecular docking analysis was performed to predict the potential interaction between melittin and the DENV envelope (E) protein. The three-dimensional structure of melittin was predicted using the AlphaFold Protein Structure Database, whereas the crystal structure of the DENV-2 envelope protein was retrieved from the Protein Data Bank (PDB ID: 3UZV) [66]. Protein–protein docking was performed using the ZDOCK v3.0.2 program with default parameters. Docking poses were ranked according to the ZDOCK scoring function, and the highest-scoring complex was selected for interaction analysis. Based on a previous study identifying Gly385 as a critical residue involved in ligand interaction with the DENV envelope protein [67], docking models involving this region were further examined. Protein–protein interactions were subsequently visualized and analyzed to identify putative binding residues and interaction types, including hydrogen bonds, hydrophobic interactions, and van der Waals contacts.

### 4.13. EC_50_, CC_50_, and Selectivity Index Analysis

The 50% effective concentration (EC_50_) and 50% cytotoxic concentration (CC_50_) were calculated using the log (inhibitor) vs. normalized response—Variable slope nonlinear regression model in GraphPad Prism version 8 (GraphPad Software, Inc., La Jolla, CA, USA). The EC_50_ values were determined from the antiviral activity measured by the focus-forming unit (FFU) reduction assay, whereas the CC_50_ values were obtained from the 72-h cell viability assay. The corresponding 95% confidence intervals (95% CI) were calculated by GraphPad Prism. The selectivity index (SI) was calculated as the ratio of CC_50_ to EC_50_ (SI = CC_50_/EC_50_).

### 4.14. Statistical Analysis

Data are presented as the mean ± SEM. FFU reduction and crystal violet assays were performed using three independent biological replicates, each with duplicate technical replicates, whereas cell-based ELISA was performed using four independent biological replicates with single-well measurements. Statistical analyses were performed using GraphPad Prism version 8 (GraphPad Software, Inc., La Jolla, CA, USA). Comparisons among multiple groups were performed using one-way analysis of variance (ANOVA) followed by Tukey’s multiple comparisons test. A *p*-value < 0.05 was considered statistically significant.

## 5. Conclusions

In the present study, melittin and *Clinacanthus nutans* extract demonstrated significant antiviral activity against DENV-2, with combination treatment enhancing antiviral efficacy while improving cell viability and attenuating melittin-associated cytotoxicity. However, the present findings are limited to an in vitro model, and the precise antiviral mechanisms underlying these effects remain to be experimentally established. In addition, the safety and toxicity profile of melittin requires further evaluation before its translational potential can be considered. Collectively, these findings support the potential of combining melittin with *C. nutans* as a promising antiviral strategy and provide a basis for further mechanistic and preclinical investigation against DENV infection. 

## Figures and Tables

**Table 1:** Half-maximal cytotoxic concentration (CC_50_) of Melittin in Vero Cells.

Time After Treatment	CC_50_ (µg/mL)
24 h	3.444
48 h	4.867
72 h	4.303

**Table 2:** Synergy analysis of melittin and *C. nutans* extract.

Melittin (µg/mL)	*C. nutans* (µg/mL)	ΔBliss	Synergy Factor	Interpretation
1.25	7.8125	0.257	19.615	Synergistic
1.25	15.625	0.190	12.588	Synergistic

**Table 3:** Putatively identified bioactive compounds in *C. nutans* extract detected by LC-MS/MS.

Name	Structural/Molecular Formula	Antiviral Activities	Anti-Apoptosis	Cell Proliferation
Acteoside; Verbascoside; Kusaginin	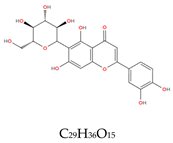	HSV-1 and HSV-2 [26] RSV [27] DENV-2 [28]	Inhibited IL-6, IL-12, TNF-α, IFN-γ, and IL-1β [29] caspase 3 [30]	Promoted cell proliferation [31]
Homoorientin (Isoorientin)	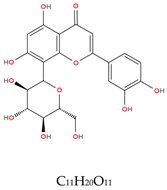	-	Inhibited ROS [32] inhibited PI3K/Akt [33]	Inhibited cell proliferation [32]
Orientin	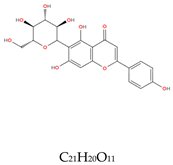	-	Inhibited Bax, Cytochrome c and Caspase-3 And Induced Bcl-2 [34]	Inhibited cell proliferation [35]
Isovitexin	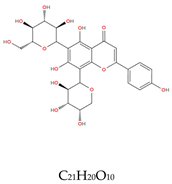	HSV-1, HSV-2, HAV, Coxsackievirus B4 and SARS-CoV-2 [36]	Inhibited SHP2, ROS, Bax and caspase-3 [37]	Inhibited cell proliferation [37]
Schaftoside	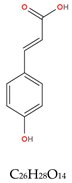	SARS-CoV-2 [38]	Inhibited Bax, Caspase-3 and induced Bcl-2 [39]	-
p-Coumaric acid	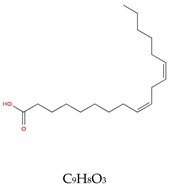	Influenza virus [40]	-	Promoted cell proliferation [41]
Linoleic acid	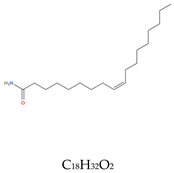	ZiKa virus, DENV-2, HSV-1, influenza virus and SARS-CoV-2 [42]	Inhibited NF-κB [43]	Inhibited cell proliferation [44]
Oleamide	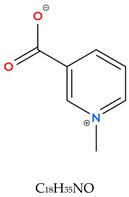	-	-	Inhibited cell proliferation [45]
Trigonelline	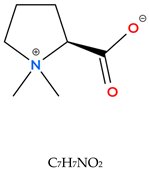	HSV-1 [46]	Inhibited Bax [46]	Inhibited cell proliferation [47]
Stachydrine	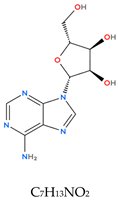	-	Inhibited NF-kB, PI3K/Akt, MAPK [48]	Inhibited cell proliferation [48]
Adenosine	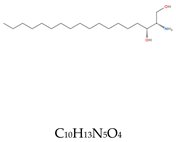	SARS-CoV-2 [49]	Inhibited A2A and induce A3 [50]	Promoted cell proliferation and inhibited cell proliferation [50]
D-erythro-Sphinganine	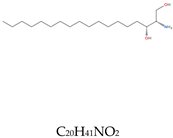	-	-	Inhibited cell proliferation [51]

## Data Availability

The raw data supporting the conclusions of this article will be made available by the authors on request.

## Supplementary Materials

The following supporting information can be downloaded at https://www.mdpi.com/article/10.3390/ijms27177782/s1.

## Abbreviations

The following abbreviations are used in this manuscript:

ANOVA	Analysis of variance
CC_50_	Cytotoxic concentration
CI	Confidence interval
DENV	Dengue virus
EC_50_	50% effective concentration
ELISA	Enzyme-linked immunosorbent
FFU	Focus forming unit
HPLC	High-performance liquid chromatography
IFA	Immunofluorescence assay
SEM	Standard error of the mean
SI	Selectivity index
SF	Synergy factor

## References

[1] World Health Organization Global Dengue Surveillance. https://worldhealthorg.shinyapps.io/dengue_global/.

[2] Guzman M.G., Harris E. (2015). Dengue. Lancet.

[3] Bhatt S., Gething P.W., Brady O.J., Messina J.P., Farlow A.W., Moyes C.L., Drake J.M., Brownstein J.S., Hoen A.G., Sankoh O. (2013). The global distribution and burden of dengue. Nature.

[4] Simmons C.P., Farrar J.J., van Vinh Chau N., Wills B. (2012). Dengue. N. Engl. J. Med..

[5] Martina B.E., Koraka P., Osterhaus A.D. (2009). Dengue virus pathogenesis: An integrated view. Clin. Microbiol. Rev..

[6] Lindenbach B.D., Thiel H.J., Rice C.M., Knipe D.M., Howley P.M. (2007). Flaviviridae: The Viruses and Their Replication. Fields Virology.

[7] Mukhopadhyay S., Kuhn R.J., Rossmann M.G. (2005). A structural perspective of the flavivirus life cycle. Nat. Rev. Microbiol..

[8] Perera-Lecoin M., Meertens L., Carnec X., Amara A. (2013). Flavivirus entry receptors: An update. Viruses.

[9] Cruz-Oliveira C., Freire J.M., Conceicao T.M., Higa L.M., Castanho M.A., Da Poian A.T. (2015). Receptors and routes of dengue virus entry into the host cells. FEMS Microbiol. Rev..

[10] Alphey L., McKemey A., Nimmo D., Oviedo M.N., Lacroix R., Matzen K., Beech C. (2016). Genetic Control of *Aedes* Mosquitoes. Global Health Impacts of Vector-Borne Diseases: Workshop Summary.

[11] Hoffmann A.A., Montgomery B.L., Popovici J., Iturbe-Ormaetxe I., Johnson P.H., Muzzi F., Greenfield M., Durkan M., Leong Y.S., Dong Y. (2011). Successful establishment of Wolbachia in Aedes populations to suppress dengue transmission. Nature.

[12] Hadinegoro S.R., Arredondo-Garcia J.L., Capeding M.R., Deseda C., Chotpitayasunondh T., Dietze R., Muhammad Ismail H.I., Reynales H., Limkittikul K., Rivera-Medina D.M. (2015). Efficacy and Long-Term Safety of a Dengue Vaccine in Regions of Endemic Disease. N. Engl. J. Med..

[13] Sridhar S., Luedtke A., Langevin E., Zhu M., Bonaparte M., Machabert T., Savarino S., Zambrano B., Moureau A., Khromava A. (2018). Effect of Dengue Serostatus on Dengue Vaccine Safety and Efficacy. N. Engl. J. Med..

[14] Biswal S., Reynales H., Saez-Llorens X., Lopez P., Borja-Tabora C., Kosalaraksa P., Sirivichayakul C., Watanaveeradej V., Rivera L., Espinoza F. (2019). Efficacy of a Tetravalent Dengue Vaccine in Healthy Children and Adolescents. N. Engl. J. Med..

[15] Kirkpatrick B.D., Whitehead S.S., Pierce K.K., Tibery C.M., Grier P.L., Hynes N.A., Larsson C.J., Sabundayo B.P., Talaat K.R., Janiak A. (2016). The live attenuated dengue vaccine TV003 elicits complete protection against dengue in a human challenge model. Sci. Transl. Med..

[16] Pourzangiabadi M., Najafi H., Fallah A., Goudarzi A., Pouladi I. (2025). Dengue virus: Etiology, epidemiology, pathobiology, and developments in diagnosis and control—A comprehensive review. Infect. Genet. Evol..

[17] Wehbe R., Frangieh J., Rima M., El Obeid D., Sabatier J.M., Fajloun Z. (2019). Bee Venom: Overview of Main Compounds and Bioactivities for Therapeutic Interests. Molecules.

[18] Raghuraman H., Chattopadhyay A. (2007). Melittin: A membrane-active peptide with diverse functions. Biosci. Rep..

[19] Memariani H., Memariani M., Moravvej H., Shahidi-Dadras M. (2020). Melittin: A venom-derived peptide with promising anti-viral properties. Eur. J. Clin. Microbiol. Infect. Dis..

[20] Hider R.C. (1988). Honeybee venom: A rich source of pharmacologically active peptides. Endeavour.

[21] Sattayawat P., Kaewkod T., Thongyim S., Chiawpanit C., Wutti-In Y., Thepmalee C., Tragoolpua Y., Disayathanoowat T., Panya A. (2025). A Comparative Study of Melittins from Apis florea and Apis mellifera as Cytotoxic Agents Against Non-Small Cell Lung Cancer (NSCLC) Cells and Their Combination with Gefitinib. Int. J. Mol. Sci..

[22] Thongyim S., Chiangchin S., Pandith H., Tragoolpua Y., Jangsutthivorawat S., Panya A. (2023). Anti-Inflammatory Activity of Glyceryl 1,3-Distearate Identified from *Clinacanthus nutans* Extract against Bovine Mastitis Pathogens. Antibiotics.

[23] Alam A., Ferdosh S., Ghafoor K., Hakim A., Juraimi A.S., Khatib A., Sarker Z.I. (2016). *Clinacanthus nutans*: A review of the medicinal uses, pharmacology and phytochemistry. Asian Pac. J. Trop. Med..

[24] Jantakee K., Panwong S., Sattayawat P., Sumankan R., Saengmuang S., Choowongkomon K., Panya A. (2024). *Clinacanthus nutans* (Burm. f.) Lindau Extract Inhibits Dengue Virus Infection and Inflammation in the Huh7 Hepatoma Cell Line. Antibiotics.

[25] Panya A., Pundith H., Thongyim S., Kaewkod T., Chitov T., Bovonsombut S., Tragoolpua Y. (2020). Antibiotic-Antiapoptotic Dual Function of *Clinacanthus nutans* (Burm. f.) Lindau Leaf Extracts against Bovine Mastitis. Antibiotics.

[26] Martins F.O., Esteves P.F., Mendes G.S., Barbi N.S., Menezes F.S., Romanos M.T. (2009). Verbascoside isolated from Lepechinia speciosa has inhibitory activity against HSV-1 and HSV-2 in vitro. Nat. Prod. Commun..

[27] Chathuranga K., Kim M.S., Lee H.C., Kim T.H., Kim J.H., Gayan Chathuranga W.A., Ekanayaka P., Wijerathne H., Cho W.K., Kim H.I. (2019). Anti-Respiratory Syncytial Virus Activity of Plantago asiatica and Clerodendrum trichotomum Extracts In Vitro and In Vivo. Viruses.

[28] Brandao G.C., Kroon E.G., Souza D.E., Souza Filho J.D., Oliveira A.B. (2013). Chemistry and Antiviral Activity of *Arrabidaea pulchra* (Bignoniaceae). Molecules.

[29] Qiao Z., Tang J., Wu W., Tang J., Liu M. (2019). Acteoside inhibits inflammatory response via JAK/STAT signaling pathway in osteoarthritic rats. BMC Complement. Altern. Med..

[30] Khorashadizadeh N., Neamati A., Moshiri M., Etemad L. (2022). Verbascoside inhibits paraquate-induced pulmonary toxicity via modulating oxidative stress, inflammation, apoptosis and DNA damage in A549 cell. Drug Chem. Toxicol..

[31] Yoou M.S., Kim H.M., Jeong H.J. (2015). Acteoside attenuates TSLP-induced mast cell proliferation via down-regulating MDM2. Int. Immunopharmacol..

[32] Yuan L., Wang J., Wu W., Liu Q., Liu X. (2016). Effect of isoorientin on intracellular antioxidant defence mechanisms in hepatoma and liver cell lines. Biomed. Pharmacother..

[33] Yuan L., Wang J., Xiao H., Xiao C., Wang Y., Liu X. (2012). Isoorientin induces apoptosis through mitochondrial dysfunction and inhibition of PI3K/Akt signaling pathway in HepG2 cancer cells. Toxicol. Appl. Pharmacol..

[34] Fu X.C., Wang M.W., Li S.P., Wang H.L. (2006). Anti-apoptotic effect and the mechanism of orientin on ischaemic/reperfused myocardium. J. Asian Nat. Prod. Res..

[35] Guo Q., Tian X., Yang A., Zhou Y., Wu D., Wang Z. (2014). Orientin in *Trollius chinensis* Bunge inhibits proliferation of HeLa human cervical carcinoma cells by induction of apoptosis. Monatshefte Für Chem.-Chem. Mon..

[36] Mohammed H.S., Khalifa S.M., Taha E.F.S., Ahmed A.H., Eissa I.H., Metwaly A.M., Marzouk M. (2026). Broad-spectrum antiviral potential of vitexin and isovitexin from Jatropha integerrima: In vitro cytoprotective effects and in silico insights. Naunyn Schmiedebergs Arch. Pharmacol..

[37] Zhang Y., Qi Z., Wang W., Wang L., Cao F., Zhao L., Fang X. (2021). Isovitexin Inhibits Ginkgolic Acids-Induced Inflammation Through Downregulating SHP2 Activation. Front. Pharmacol..

[38] Yi Y., Zhang M., Xue H., Yu R., Bao Y.O., Kuang Y., Chai Y., Ma W., Wang J., Shi X. (2022). Schaftoside inhibits 3CL^pro^ and PL^pro^ of SARS-CoV-2 virus and regulates immune response and inflammation of host cells for the treatment of COVID-19. Acta Pharm. Sin. B.

[39] Limpanich N., Chayapakdee P., Mekawan K., Thongyim S., Yongsawas R., Khamwong P., Tragoolpua Y., Kaewkod T., Jangsutthivorawat S., Jungklang J. (2025). Integrative Wound-Healing Effects of *Clinacanthus nutans* Extract and Schaftoside Through Anti-Inflammatory, Endothelial-Protective, and Antiviral Mechanisms. Int. J. Mol. Sci..

[40] Kai H., Obuchi M., Yoshida H., Watanabe W., Tsutsumi S., Park Y.K., Matsuno K., Yasukawa K., Kurokawa M. (2014). In vitro and in vivo anti-influenza virus activities of flavonoids and related compounds as components of Brazilian propolis (AF-08). J. Funct. Foods.

[41] Tehami W., Nani A., Khan N.A., Hichami A. (2023). New Insights Into the Anticancer Effects of p-Coumaric Acid: Focus on Colorectal Cancer. Dose Response.

[42] Feng Y., Yang Y., Zou S., Qiu S., Yang H., Hu Y., Lin G., Yao X., Liu S., Zou M. (2023). Identification of alpha-linolenic acid as a broad-spectrum antiviral against zika, dengue, herpes simplex, influenza virus and SARS-CoV-2 infection. Antivir. Res..

[43] Lee D.K., Choi K.H., Oh J.N., Kim S.H., Lee M., Jeong J., Choe G.C., Lee C.K. (2022). Linoleic acid reduces apoptosis via NF-kappaB during the in vitro development of induced parthenogenic porcine embryos. Theriogenology.

[44] Kemp M.Q., Jeffy B.D., Romagnolo D.F. (2003). Conjugated linoleic acid inhibits cell proliferation through a p53-dependent mechanism: Effects on the expression of G1-restriction points in breast and colon cancer cells. J. Nutr..

[45] Torres-Roman A.L., Ortega-Gomez A., Reyes-Soto C.Y., Aparicio-Trejo O.E., Cuevas-Lopez B., Garcia-Arroyo F.E., Ruiz-Garcia E., Matus-Santos J.A., Ferrer B., Aschner M. (2026). The Anti-proliferative Effects of Anandamide and Oleamide in Glioblastoma Cell Lines Recruit Mitochondrial and PPAR-gamma Receptor Modulation. Neurochem. Res..

[46] Ozcelik B., Kartal M., Orhan I. (2011). Cytotoxicity, antiviral and antimicrobial activities of alkaloids, flavonoids, and phenolic acids. Pharm. Biol..

[47] Dhayalan S., Ramarajyam G., Mohanprasanth A., Muruhesan D., Rajendiran S., Arjunan S. (2026). Trigonelline suppresses the tumor progression via STAT2 signalling pathway in non-Small cell lung carcinoma. Med. Oncol..

[48] He Z., Li P., Liu P., Xu P. (2024). Exploring stachydrine: From natural occurrence to biological activities and metabolic pathways. Front. Plant Sci..

[49] Monticone G., Huang Z., Hewins P., Cook T., Mirzalieva O., King B., Larter K., Miller-Ensminger T., Sanchez-Pino M.D., Foster T.P. (2024). Novel immunomodulatory properties of adenosine analogs promote their antiviral activity against SARS-CoV-2. EMBO Rep..

[50] Merighi S., Mirandola P., Milani D., Varani K., Gessi S., Klotz K.N., Leung E., Baraldi P.G., Borea P.A. (2002). Adenosine receptors as mediators of both cell proliferation and cell death of cultured human melanoma cells. J. Investig. Dermatol..

[51] Lachaud C., Da Silva D., Cotelle V., Thuleau P., Xiong T.C., Jauneau A., Briere C., Graziana A., Bellec Y., Faure J.D. (2010). Nuclear calcium controls the apoptotic-like cell death induced by d-erythro-sphinganine in tobacco cells. Cell Calcium.

[52] Lee G., Bae H. (2016). Anti-Inflammatory Applications of Melittin, a Major Component of Bee Venom: Detailed Mechanism of Action and Adverse Effects. Molecules.

[53] Guha S., Ferrie R.P., Ghimire J., Ventura C.R., Wu E., Sun L., Kim S.Y., Wiedman G.R., Hristova K., Wimley W.C. (2021). Applications and evolution of melittin, the quintessential membrane active peptide. Biochem. Pharmacol..

[54] Panya A., Bangphoomi K., Choowongkomon K., Yenchitsomanus P.T. (2014). Peptide inhibitors against dengue virus infection. Chem. Biol. Drug Des..

[55] Panya A., Sawasdee N., Junking M., Srisawat C., Choowongkomon K., Yenchitsomanus P.T. (2015). A peptide inhibitor derived from the conserved ectodomain region of DENV membrane (M) protein with activity against dengue virus infection. Chem. Biol. Drug Des..

[56] Panya A., Yongpitakwattana P., Budchart P., Sawasdee N., Krobthong S., Paemanee A., Roytrakul S., Rattanabunyong S., Choowongkomon K., Yenchitsomanus P.T. (2019). Novel bioactive peptides demonstrating anti-dengue virus activity isolated from the Asian medicinal plant Acacia Catechu. Chem. Biol. Drug Des..

[57] Fosgerau K., Hoffmann T. (2015). Peptide therapeutics: Current status and future directions. Drug Discov. Today.

[58] Craik D.J., Fairlie D.P., Liras S., Price D. (2013). The future of peptide-based drugs. Chem. Biol. Drug Des..

[59] Vaughn D.W., Green S., Kalayanarooj S., Innis B.L., Nimmannitya S., Suntayakorn S., Endy T.P., Raengsakulrach B., Rothman A.L., Ennis F.A. (2000). Dengue viremia titer, antibody response pattern, and virus serotype correlate with disease severity. J. Infect. Dis..

[60] Jessie K., Fong M.Y., Devi S., Lam S.K., Wong K.T. (2004). Localization of dengue virus in naturally infected human tissues, by immunohistochemistry and in situ hybridization. J. Infect. Dis..

[61] Low J.G., Ooi E.E., Tolfvenstam T., Leo Y.S., Hibberd M.L., Ng L.C., Lai Y.L., Yap G.S., Li C.S., Vasudevan S.G. (2006). Early Dengue infection and outcome study (EDEN)—study design and preliminary findings. Ann. Acad. Med. Singap..

[62] Wilder-Smith A., Ooi E.E., Horstick O., Wills B. (2019). Dengue. Lancet.

[63] Thongyim S., Sattayawat P., Pongamornkul W., Jangsutthivorawat S., Tragoolpua Y., Panya A. (2025). Schaftoside contributed to anti-inflammatory activity of *Clinacanthus nutans* extract in lipopolysaccharide-induced RAW 264.7 cells. Front. Pharmacol..

[64] Zhao W., Sachsenmeier K., Zhang L., Sult E., Hollingsworth R.E., Yang H. (2014). A New Bliss Independence Model to Analyze Drug Combination Data. J. Biomol. Screen.

[65] Demidenko E., Miller T.W. (2019). Statistical determination of synergy based on Bliss definition of drugs independence. PLoS ONE.

[66] Cockburn J.J., Navarro Sanchez M.E., Fretes N., Urvoas A., Staropoli I., Kikuti C.M., Coffey L.L., Arenzana Seisdedos F., Bedouelle H., Rey F.A. (2012). Mechanism of dengue virus broad cross-neutralization by a monoclonal antibody. Structure.

[67] Agrawal P., Arya H., Senthil Kumar G. (2024). Structure-based identification of small-molecule inhibitors that target the DIII domain of the Dengue virus glycoprotein E pan-serotypically. PLoS ONE.

