# Supplementary material for: Combination of Melittin and Clinacanthus nutans Extract Enhances Antiviral Efficacy Against Dengue Virus Serotype 2 Infection
Source: Int J Mol Sci. 2026 Aug 31;27(17):7782. doi: 10.3390/ijms27177782 (PMC13565815; doi:10.3390/ijms27177782)
Supplement: Supplementary file 1 [file ijms-27-07782-s001.zip › ijms-4436493-supplementary.pdf]

**Supplementary Data**

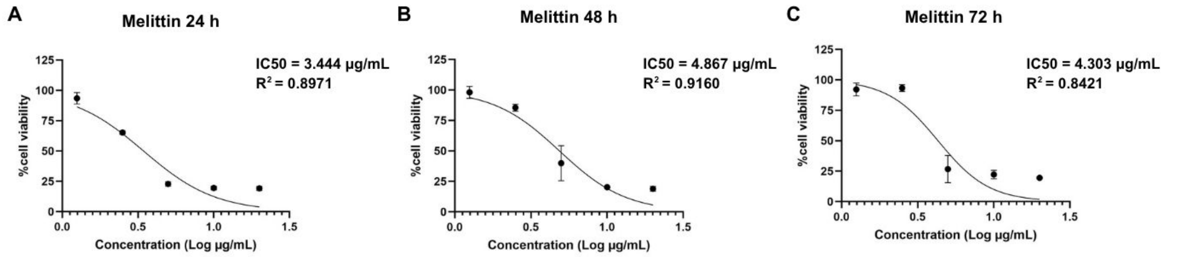


**Supplementary Figure S1. Cytotoxicity of melittin in Vero cells.** Vero cells were treated with serial concentrations of melittin for 24 (A), 48 (B), and 72 h (C). Cell viability was determined by the Crystal Violet staining assay. Dose–response curves were generated by nonlinear regression to determine the CC50 values.

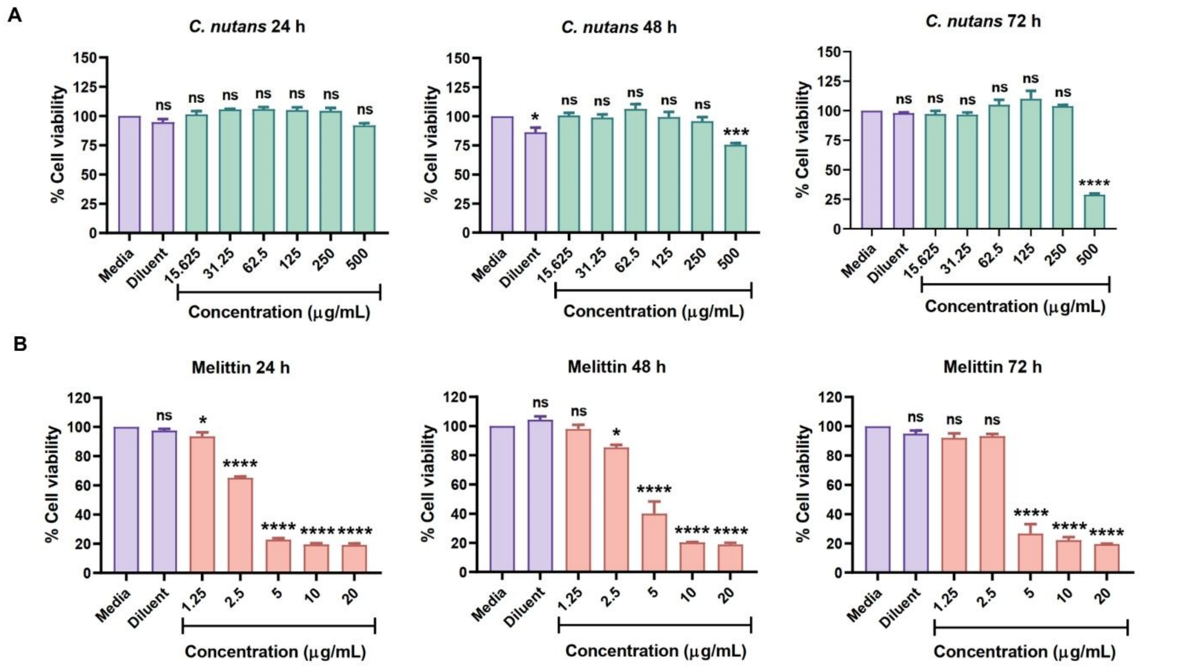


**Supplementary Figure S2. Cytotoxicity of *C. nutans* extract and melittin in Vero cells.** Vero cells were treated with increasing concentrations of *C. nutans* extract (15.625–500 µg/mL) (A) or melittin (1.25–20 µg/mL) (B) for 24, 48, and 72 h. Cell viability was determined using the Crystal Violet staining assay and expressed as the percentage of viable cells relative to the untreated control. Data are presented as mean ± SEM (N = 3). ns, not significant; $p < 0.05$, **p** < 0.01, ***p*** < 0.001, **p** < 0.0001 vs. the media control.

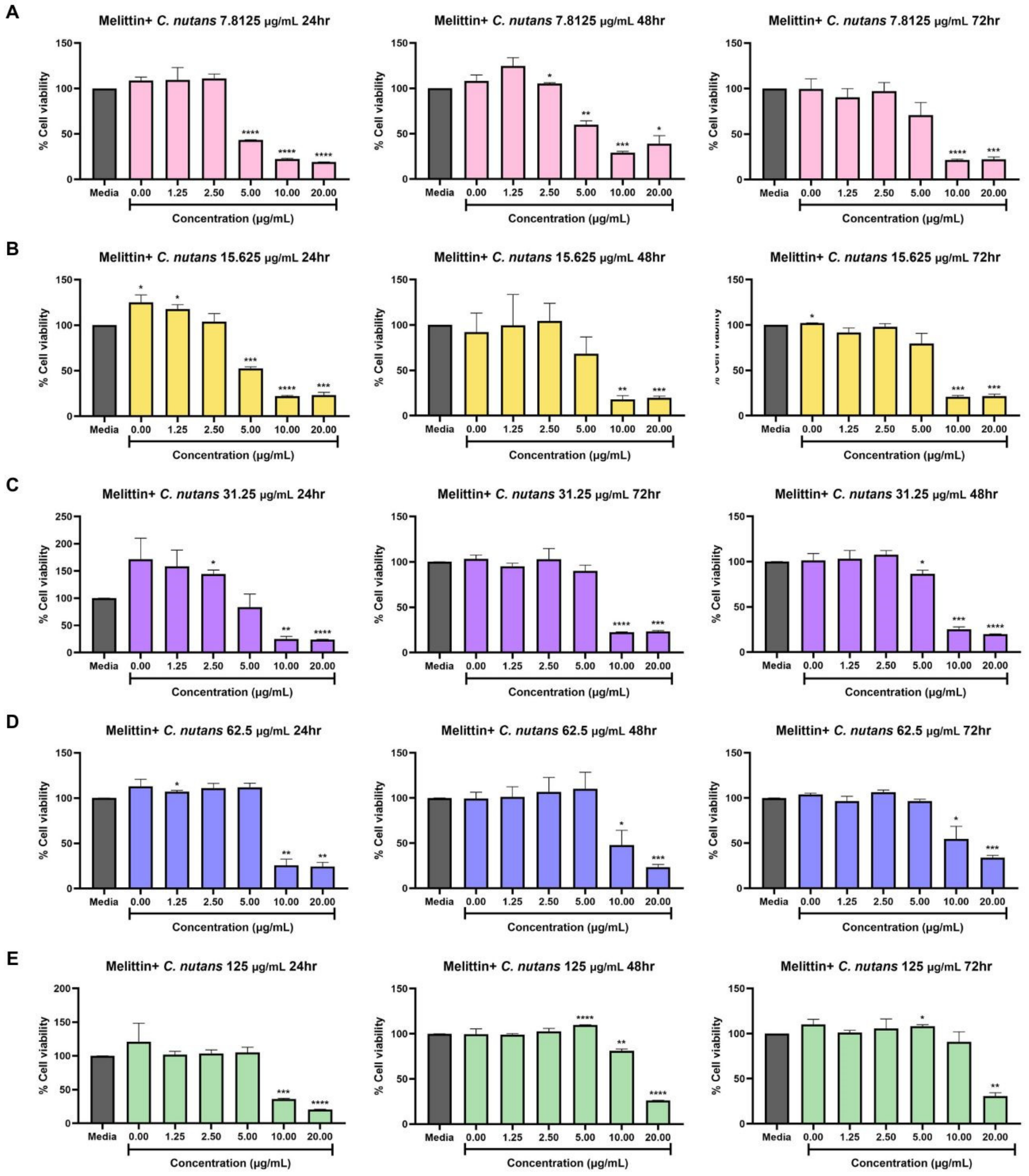


**Supplementary Figure S3. Cytotoxicity of combined melittin and *C. nutans* extract in Vero cells.** Vero cells were treated with increasing concentrations of melittin (0–20 µg/mL) in combination with *C. nutans* extract at fixed concentrations of 7.8125 (A), 15.625 (B), 31.25 (C), 62.5 (D), and 125 µg/mL (E) for 24, 48, and 72 h. Cell viability was determined using the Crystal Violet staining assay and expressed as the percentage of viable cells relative to the untreated media control. Data are presented as mean ± SEM (N = 3). *(* $p < 0.05$, ** $p < 0.01$, *** $p < 0.001$, **** $p < 0.0001$ vs. the media control.)*

**Supplementary Table S1** List of bioactive compounds

| No | Name | Molecular formula | Found At Mass |
|---|---|---|---|
| 1 | Isovitexin | $C_{21}H_{20}O_{10}$ | 431.1113 (Negative ion mode)<br>433.1179 (Positive ion mode) |
| 2 | Phenylalanine | $C_9H_{11}NO_2$ | 164.0755 (Negative ion mode)<br>166.0876 (Positive ion mode) |
| 3 | L-Tryptophan | $C_{11}H_{12}N_2O_2$ | 203.0897 (Negative ion mode)<br>205.0984 (Positive ion mode) |
| 4 | Homoorientin (Isoorientin) | $C_{21}H_{20}O_{11}$ | 447.0956 (Negative ion mode)<br>449.1086 (Positive ion mode) |
| 5 | Pipecolinic acid | $C_6H_{11}NO_2$ | 128.0345 (Negative ion mode) |
| 6 | Luteolin-7-O-β-D-glucoside | $C_{21}H_{19}O_{11}$ | 561.0914 (Negative ion mode) |
| 7 | Orientin | $C_{21}H_{20}O_{11}$ | 483.0725 (Negative ion mode) |
| 8 | Acteoside; Verbascoside; Kusaginin | $C_{29}H_{36}O_{15}$ | 623.2039 (Negative ion mode) |
| 9 | p-Coumaric acid | $C_9H_8O_3$ | 163.0392 (Negative ion mode) |
| 10 | Linoleic acid | $C_{18}H_{32}O_2$ | 279.2401 (Negative ion mode) |
| 11 | Adenine | $C_5H_5N_5$ | 134.0470 (Negative ion mode) |
| 12 | Phenprobamate | $C_{10}H_{13}NO_2$ | 164.0756 (Negative ion mode) |
| 13 | 8-epiloganic acid | $C_{16}H_{24}O_{10}$ | 375.1412 (Negative ion mode) |
| 14 | *Proline* | $C_5H_9NO_2$ | 116.0708 (Positive ion mode) |
| 15 | *3.alpha.-Mannobiose* | $C_{12}H_{22}O_{11}$ | 360.1521 (Positive ion mode) |
| 16 | *Trigonelline* | $C_7H_7NO_2$ | 138.0549 (Positive ion mode) |
| 17 | *Betaine* | $C_5H_{11}NO_2$ | 118.0950 (Positive ion mode) |
| 18 | *Stachydrine* | $C_7H_{13}NO_2$ | 144.1106 (Positive ion mode) |
| 19 | *Nicotinic acid* | $C_6H_5NO_2$ | 124.0394 (Positive ion mode) |
| 20 | *Adenosine* | $C_{10}H_{13}N_5O_4$ | 268.1044 (Positive ion mode) |
| 21 | *Glucosylvitexin* | $C_{27}H_{30}O_{15}$ | 595.1682 (Positive ion mode) |
| 22 | *Schaftoside* | $C_{26}H_{28}O_{14}$ | 565.1675 (Positive ion mode) |
| 23 | *1H-Indole-4-carboxaldehyde* | $C_9H_7NO$ | 146.0602 (Positive ion mode) |
| 24 | *Oleamide* | $C_{18}H_{35}NO$ | 282.2816 (Positive ion mode) |
| 25 | *Indole-6-carboxaldehyde* | $C_9H_7NO$ | 146.0606 (Positive ion mode) |
| 26 | *Ethyl .beta.-carboline-3-carboxylate* | $C_{14}H_{12}N_2O_2$ | 241.0986 (Positive ion mode) |
| 27 | *1-Palmitoyl-sn-glycero-3-phosphocholine* | $C_{24}H_{50}NO_7P$ | 496.3425 (Positive ion mode) |
| 28 | *D-erythro-Sphinganine* | $C_{18}H_{39}NO_2$ | 302.3066 (Positive ion mode) |
| 29 | *1-O-Hexadecyl-2-O-acetyl-sn-glyceryl-3-phosphorylcholine* | $C_{26}H_{54}NO_7P$ | 524.3716 (Positive ion mode) |

**Supplementary Table S2.** Quality control and phytochemical characterization of the *Clinacanthus nutans* extract from previous study (Cited to Thongyim, S.; Sattayawat, P.; Pongamornkul, W.; Jangsutthivorawat, S.; Tragoolpua, Y.; Panya, A. Schaftoside contributed to anti-inflammatory activity of Clinacanthus nutans extract in lipopolysaccharide-induced RAW 264.7 cells. *Front Pharmacol* **2025**, *16*, 1584620, doi:10.3389/fphar.2025.1584620.)

| Parameter | Results |
|---|---|
| Extraction method | 95% ethanol extraction |
| Batch | Single batch used throughout the study |
| Total phenolic content | 11.857 mg GAE/g extract |
| Major marker compounds | Schaftoside, Vitexin, Isovitexin, Orientin, Isoorientin |
